# Early volumetric, perfusion, and diffusion MRI changes after mutant isocitrate dehydrogenase (IDH) inhibitor treatment in IDH1-mutant gliomas

**DOI:** 10.1093/noajnl/vdac124

**Published:** 2022-08-04

**Authors:** Nicholas S Cho, Akifumi Hagiwara, Blaine S C Eldred, Catalina Raymond, Chencai Wang, Francesco Sanvito, Albert Lai, Phioanh Nghiemphu, Noriko Salamon, Lori Steelman, Islam Hassan, Timothy F Cloughesy, Benjamin M Ellingson

**Affiliations:** Medical Scientist Training Program, David Geffen School of Medicine, University of California, Los Angeles, Los Angeles, CA, USA; UCLA Brain Tumor Imaging Laboratory (BTIL), Center for Computer Vision and Imaging Biomarkers, University of California, Los Angeles, Los Angeles, CA, USA; Department of Bioengineering, Henry Samueli School of Engineering and Applied Science, University of California, Los Angeles, Los Angeles, CA, USA; Department of Radiological Sciences, David Geffen School of Medicine, University of California, Los Angeles, Los Angeles, CA, USA; UCLA Brain Tumor Imaging Laboratory (BTIL), Center for Computer Vision and Imaging Biomarkers, University of California, Los Angeles, Los Angeles, CA, USA; Department of Radiology, Juntendo University School of Medicine, Tokyo, Japan; Department of Radiological Sciences, David Geffen School of Medicine, University of California, Los Angeles, Los Angeles, CA, USA; UCLA Neuro-Oncology Program, David Geffen School of Medicine, University of California, Los Angeles, Los Angeles, CA, USA; UCLA Brain Tumor Imaging Laboratory (BTIL), Center for Computer Vision and Imaging Biomarkers, University of California, Los Angeles, Los Angeles, CA, USA; Department of Radiological Sciences, David Geffen School of Medicine, University of California, Los Angeles, Los Angeles, CA, USA; UCLA Brain Tumor Imaging Laboratory (BTIL), Center for Computer Vision and Imaging Biomarkers, University of California, Los Angeles, Los Angeles, CA, USA; Department of Radiological Sciences, David Geffen School of Medicine, University of California, Los Angeles, Los Angeles, CA, USA; UCLA Brain Tumor Imaging Laboratory (BTIL), Center for Computer Vision and Imaging Biomarkers, University of California, Los Angeles, Los Angeles, CA, USA; Unit of Radiology, Department of Clinical, Surgical, Diagnostic, and Pediatric Sciences, University of Pavia, Pavia, Italy; Department of Radiological Sciences, David Geffen School of Medicine, University of California, Los Angeles, Los Angeles, CA, USA; UCLA Neuro-Oncology Program, David Geffen School of Medicine, University of California, Los Angeles, Los Angeles, CA, USA; UCLA Neuro-Oncology Program, David Geffen School of Medicine, University of California, Los Angeles, Los Angeles, CA, USA; Department of Radiological Sciences, David Geffen School of Medicine, University of California, Los Angeles, Los Angeles, CA, USA; Servier Pharmaceuticals, LLC, Boston, MA, USA; Servier Pharmaceuticals, LLC, Boston, MA, USA; UCLA Neuro-Oncology Program, David Geffen School of Medicine, University of California, Los Angeles, Los Angeles, CA, USA; UCLA Brain Tumor Imaging Laboratory (BTIL), Center for Computer Vision and Imaging Biomarkers, University of California, Los Angeles, Los Angeles, CA, USA; Department of Bioengineering, Henry Samueli School of Engineering and Applied Science, University of California, Los Angeles, Los Angeles, CA, USA; Department of Neurosurgery, David Geffen School of Medicine, University of California, Los Angeles, Los Angeles, CA, USA; Department of Psychiatry and Biobehavioral Sciences, David Geffen School of Medicine, University of California, Los Angeles, Los Angeles, CA, USA

**Keywords:** apparent diffusion coefficient, dynamic susceptibility contrast perfusion MRI, glioma, IDH-mutant, IDH inhibitor

## Abstract

**Background:**

Inhibition of the isocitrate dehydrogenase (IDH)-mutant enzyme is a novel therapeutic target in IDH-mutant gliomas. Imaging biomarkers of IDH inhibitor treatment efficacy in human IDH-mutant gliomas are largely unknown. This study investigated early volumetric, perfusion, and diffusion MRI changes in IDH1-mutant gliomas during IDH inhibitor treatment.

**Methods:**

Twenty-nine IDH1-mutant glioma patients who received IDH inhibitor and obtained anatomical, perfusion, and diffusion MRI pretreatment at 3–6 weeks (*n* = 23) and/or 2–4 months (*n* = 14) of treatment were retrospectively studied. Normalized relative cerebral blood volume (nrCBV), apparent diffusion coefficient (ADC), and fluid-attenuated inversion recovery (FLAIR) hyperintensity volume were analyzed.

**Results:**

After 3–6 weeks of treatment, nrCBV was significantly increased (*P* = .004; mean %change = 24.15%) but not FLAIR volume (*P* = .23; mean %change = 11.05%) or ADC (*P* = .52; mean %change = -1.77%). Associations between shorter progression-free survival (PFS) with posttreatment nrCBV > 1.55 (*P* = .05; median PFS, 240 vs 55 days) and increased FLAIR volume > 4 cm^3^ (*P* = .06; 227 vs 29 days) trended toward significance. After 2–4 months, nrCBV, FLAIR volume, and ADC were not significantly different from baseline, but an nrCBV increase > 0% (*P* = .002; 1121 vs 257 days), posttreatment nrCBV > 1.8 (*P* = .01; 1121 vs. 270 days), posttreatment ADC < 1.15 μm^2^/ms (*P* = .02; 421 vs 215 days), median nrCBV/ADC ratio increase > 0% (*P* = .02; 1121 vs 270 days), and FLAIR volume change > 4 cm^3^ (*P* = .03; 421 vs 226.5 days) were associated with shorter PFS.

**Conclusions:**

Increased nrCBV at 3–6 weeks of treatment may reflect transient therapeutic and/or tumor growth changes, whereas nrCBV, ADC, and FLAIR volume changes occurring at 2–4 months of treatment may more accurately reflect antitumor response to IDH inhibition.

**Key Points**

- IDH inhibition may cause an increase in vascularity as early as 3–6 weeks.
- At 2–4 months posttreatment, nrCBV, ADC, and FLAIR volume reflect PFS benefit.

**Importance of the Study**

There remains no standard of care for isocitrate dehydrogenase (IDH)-mutant gliomas. IDH inhibitors are novel, targeted therapies that have only recently been explored for human IDH-mutant gliomas, so imaging biomarkers of treatment efficacy are largely unexplored. We report that early changes in perfusion, apparent diffusion coefficient, and fluid-attenuated inversion recovery hyperintensity volume after 2–4 months of treatment are significantly associated with progression-free survival benefit following IDH inhibitor treatment response in IDH1-mutant gliomas. Together, data suggest a combination of early tumor size, vascularity, and diffusion changes may be useful for clinical interpretation of treatment response and tumor progression in IDH1-mutant gliomas treated with IDH inhibitors.

Identification of isocitrate dehydrogenase (IDH) mutation status is a key component of the 2021 World Health Organization classification of gliomas.^1^ IDH mutations are present in over 70% of grade 2–3 gliomas and are the basis of the new classification of grade 4 IDH-mutant astrocytomas, formerly known as secondary glioblastomas.^1,2^ In addition to gliomas, IDH mutations have also been identified in acute myeloid leukemia (AML),^3^ chondrosarcoma,^4^ and cholangiocarcinoma.^5^ IDH mutations occur early in tumorigenesis,^6^ and they most commonly occur in cytosolic IDH1 enzymes (>95%) and less frequently in mitochondrial IDH2 enzymes.^7^ IDH1/2 normally catalyzes the oxidative decarboxylation of isocitrate into α-ketoglutarate (α-KG).^8^ Mutations in IDH1/2 result in a gain of function as the mutant enzyme catalyzes the conversion of α-KG into the oncometabolite d-2-hydroxyglutarate (D-2-HG).^9^ Elevated D-2-HG levels cause many downstream effects, including inhibiting α-KG-dependent dioxygenases, which results in DNA hypermethylation and inhibited cellular differentiation,^10,11^ as well as decreasing levels of hypoxia-inducible factor 1α (HIF-1α) that result in reduced hypoxic signaling, proangiogenic signaling, and glycolytic capacity.^12,13^

Treatment options for patients with IDH-mutant gliomas often include maximally safe surgical tumor resection followed by chemoradiation.^14,15^ However, the treatment is non-curative, as IDH-mutant gliomas usually recur and later progress. In addition, surgery and chemoradiation can negatively impact the quality of life, so balancing quality of life with survival benefits is an important consideration during clinical care.^16–18^ Moreover, patients with IDH-mutant gliomas are considerably younger and have better prognosis than patients with IDH-wild-type gliomas.^2^ Thus, novel targeted therapies for IDH-mutant gliomas that circumvent the toxicities associated with chemoradiation may provide significant benefits for a younger patient population in terms of reduced morbidity.

Inhibition of the IDH-mutant enzyme has only recently been explored as a therapeutic target in IDH-mutant gliomas.^19–22^ Preclinical studies have shown that inhibition of mutant IDH enzymes reduced D-2-HG levels,^21^ increased glutamate levels,^21^ and promoted differentiation of glioma cells.^22^ Clinical studies of IDH inhibitors have also demonstrated tumor shrinkage effects in glioma patients^19,20^ and favorable clinical responses in patients with AML,^23–25^ cholangiocarcinoma,^26^ and chondrosarcoma.^27^ MRI techniques, including dynamic susceptibility contrast (DSC) perfusion MRI and diffusion-weighted imaging (DWI), have been valuable in the management of patients with brain tumors. For example, MRI metrics such as relative cerebral blood volume (rCBV) and apparent diffusion coefficient (ADC) have been useful in monitoring treatment response and providing predictive value in glioma patients.^28–31^ However, early MRI biomarkers of treatment efficacy in IDH-mutant gliomas following IDH inhibition remain largely unknown.

This retrospective longitudinal study explored early changes in anatomical, perfusion, and diffusion MRI in patients with IDH-mutant gliomas during treatment with IDH inhibitors as well as potential associations between MRI metrics and progression-free survival (PFS). We hypothesized that perfusion, diffusion, and volumetric MRI metrics can be early biomarkers of treatment response by IDH inhibitors in IDH-mutant gliomas.

## Methods

### Patient Selection

Patients diagnosed with IDH1-mutant gliomas who received ivosidenib (AG-120) or vorasidenib (AG-881), an inhibitor of the mutant IDH enzyme, daily off-label or as part of a clinical trial (clinicaltrials.gov: NCT03343197; NCT02481154; NCT02073994) between September 2014 and May 2021 at our institution were reviewed (see Supplementary Table 1 for IDH inhibitor treatment information). Patients with the following inclusion criteria were studied: (1) received treatment with IDH inhibitor; (2) obtained DSC-MRI, DWI, and anatomical MRI scans before treatment; (3) and at ~3–6 weeks and/or ~2–4 months after treatment initiation; (4) remained on treatment throughout scan interval. The most recent MRI study before the start of IDH inhibitor treatment was used as a baseline. Patients were excluded if there was disease progression before a scan date. Patients were excluded from survival analysis if they underwent surgical tumor resection within 6 months after IDH inhibitor start date (including patients in perioperative trial NCT03343197). PFS was defined as the time between first dose of treatment to disease progression, death, or censor date. Disease progression was assessed by the treating neuro-oncologists according to the response assessment in neuro-oncology (RANO) and RANO-low grade glioma (RANO-LGG) criteria.^32,33^*IDH1* mutation was confirmed in all patients by genomic sequencing analysis, immunohistochemistry, and/or polymerase chain reaction,^34^ and 1p/19q co-deletion status was determined using fluorescence in situ hybridization. Patient data are summarized in Table 1. All patients provided informed consent approved by our institutional review board. All analyses were done in compliance with the Health Insurance Portability and Accountability Act.

**Table 1.** Patient Data

Characteristics	All Patients (*n* = 29)	Patients Scanned at 3–6 Weeks of Treatment (*n* = 23)	Patients Scanned at 3–6 Weeks of Treatment in Survival Analysis (*n* = 12)	Patients Scanned at 2–4 Months of Treatment (*n* = 14; All in Survival Analysis)
Average age (years) ± SD	42 ± 12	43 ± 11	40 ± 10	39 ± 13
Sex (male/female)	18/11	16/7	9/3	8/6
Tumor location				
Hemisphere (left/right)	17/12	13/10	6/6	7/7
Frontal lobe	19	15	7	9
Temporal lobe	7	5	4	5
Parietal lobe	3	3	1	0
Tumor grade (2/3/4)	19/7/3	14/6/3	4/6/2	9/4/1
Number of recurrence (New/1st/2nd/3rd+)	2/12/7/8	1/9/5/8	0/2/4/6	1/4/6/3
1p/19q co-deletion status (co-deleted/non-co-deleted/N/A)	11/15/3	9/12/2	4/6/2	4/8/2
IDH inhibitor drug (AG-120/AG-881)	18/11	15/8	9/3	9/5
Median progression-free survival in days and range (days)	343 (23–1970)	302 (23–1449)	185 (23–580)	320.5 (79–1970)
Median days between pretreatment scan date and start of IDH inhibitor treatment (days)	9 (0–49)	11 (0–33)	7 (0–33)	6.5 (0–49)
Median days between posttreatment scan date and treatment start date and range (days)	N/A	27 (21–42)	31 (23–42)	113 (59–121)

Abbreviation: IDH, isocitrate dehydrogenase.

### Image Acquisition and Processing

Anatomical, DWI, and DSC perfusion MRI were obtained on 1.5T or 3T MRI scanners (Siemens Healthcare; Erlangen, Germany). Anatomical MRI, including 3D pre- and post-contrast (gadolinium-diethylenetriamine pentaacetic acid at a dose of 0.1 mmoL/kg body weight; Magnevist, Bayer Schering Pharma, Leverkusen, Germany) T1-weighted images, axial T2-weighted images, fluid-attenuated inversion recovery (FLAIR), and DWI images were collected according to the international standardized brain tumor imaging protocol.^35^ For DSC perfusion MRI, images were collected according to previously described imaging protocols.^36,37^ All DSC-MRI acquisitions covered the volume of contrast-enhancing and non-enhancing tumors.

### DSC and Image Analysis

All parameter maps were registered to the post-contrast T1-weighted images using a six-degree-of-freedom rigid transformation and a mutual information cost function using FSL software (*flirt*; Functional Magnetic Resonance Imaging of the Brain Software Library; Oxford, England). A volume of interest (VOI) was segmented on the FLAIR hyperintense tumor with guidance from NS-HGlio artificial intelligence device (Neosoma Inc, Groton, MA, https://neosomainc.com) which automatically detects and segments the tumor compartments on MRIs in combination with an in-house, semi-automated thresholding method using the Analysis of Functional NeuroImages (AFNI) software (NIMH Scientific and Statistical Computing Core; Bethesda, MD, USA; https://afni.nimh.nih.gov).^38^ DSC data were first motion-corrected using FSL (*mcflirt*, FMRIB library). rCBV maps were calculated using a previously described bidirectional contrast agent leakage correction method.^39^ Then, rCBV was normalized (nrCBV) by the mean of value of 3 spherical, intra-slice VOIs of 5-mm diameter placed in the contralateral normal-appearing white matter in the centrum semiovale superior to the lateral ventricles as similarly described in a previous study^40^ using ITK-SNAP software (http://www.itksnap.org/).^41^ Median nrCBV, median ADC (μm^2^/ms), median nrCBV/ADC ratio (median nrCBV/median ADC),^42^ and tumor volume measurements were obtained and used for subsequent analyses.

### Statistical Analysis

All calculations and analyses were performed in MATLAB (Release 2020a, MathWorks, Natick, MA) or GraphPad Prism software (Version 8.4 GraphPad Software, San Diego, CA). To visualize population-based temporal trends in MRI metrics, local polynomial regression fitting was performed on MRI metrics using all timepoints as cross-sectional analysis with a cubic function fixed at the origin using the *polyfix* MATLAB package as similarly performed in a previous study.^43^ Patients were stratified based on median PFS, and all patients were used for this illustrative analysis regardless if patients were used for formal survival analysis to provide sufficient datapoints for curve-fitting. The Shapiro-Wilk test was conducted to assess if data were normally distributed and to apply appropriate parametric or nonparametric statistical methods. For normally distributed data, one-sample *t*-test analyses were conducted to assess percentage changes in MRI metrics compared to no change. For non-normally distributed data, Wilcoxon Signed-Rank analyses were conducted to assess percentage changes in MRI metrics.

Kaplan-Meier survival curves were generated to assess relationships between posttreatment nrCBV, ADC, median nrCBV/ADC ratio, or FLAIR volume, and changes in nrCBV, ADC, median nrCBV/ADC ratio, or FLAIR volume with PFS using the log-rank test. Optimal thresholds for Kaplan-Meier curves were determined by looping through quantitative values and then calculating the Mantel-Haenszel hazard ratio (HR) and corresponding *P* values for patient stratifications resulting in at least 4 patients in a group as described previously.^44^ Cox proportional hazards regression analysis was performed on the significant MRI metrics from log-rank analyses to assess if relationships remained significant using continuous measures of MRI metrics and after controlling for clinical variables of age and tumor grade. Significance level was set at α = 0.05, and all tests were two-tailed. Multiple comparison corrections were not performed because of the limited sample size.

## Results

**Figure 1.**
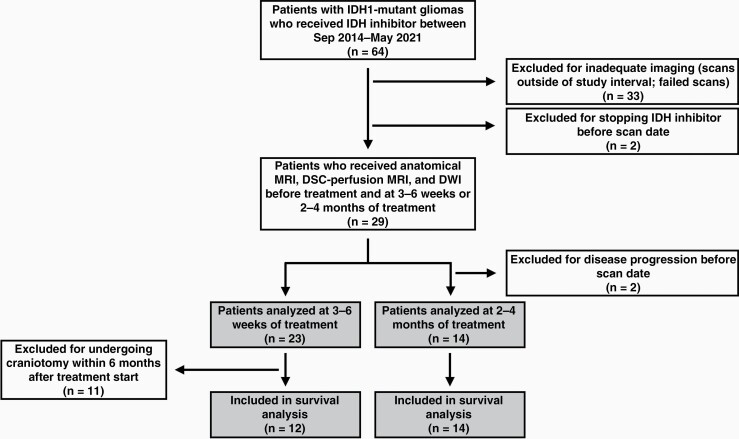
Flowchart of patient selection process. IDH, isocitrate dehydrogenase; MRI, magnetic resonance imaging; DSC, dynamic susceptibility contrast; DWI, diffusion-weighted imaging.

Among 64 patients eligible for this study, a total of 29 patients met the inclusion and exclusion criteria (Figure 1). A total of 23 patients had data available at 3–6 weeks and 14 patients had data available at 2–4 months after starting treatment. Eight patients had data at both time points. Within the patients having imaging data available 3–6 weeks after starting therapy, 11 patients were excluded for PFS analysis because they underwent craniotomy within 6 months after the start of IDH inhibitor treatment.

**Figure 2.**
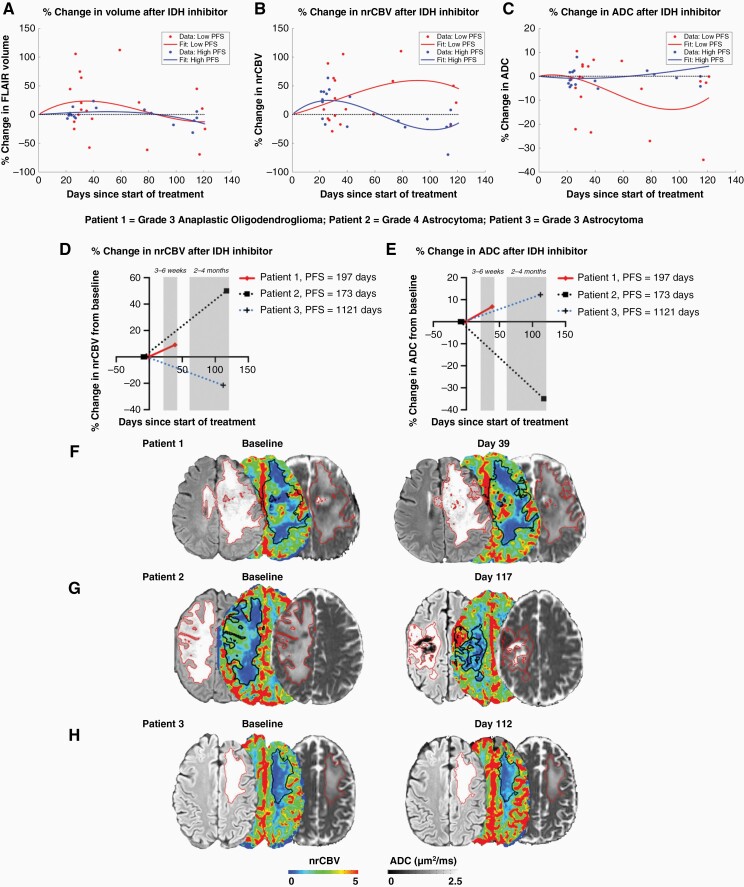
Temporal trends of FLAIR volume, nrCBV, and ADC changes following IDH inhibitor and three representative cases. Local polynomial regression trend lines (A–C) depict an early rise in nrCBV following IDH inhibitor across patients followed by increased nrCBV at later timepoints associated for the low PFS group. ADC differences appear at later timepoints with the low PFS group exhibiting decreased ADC. FLAIR volume also generally increases early in treatment. Patient 1 (F) is a grade 3 anaplastic oligodendroglioma with a large increase in nrCBV after day 39 of AG-120 treatment and presented with a PFS of around 197 days. For the 2–4-month timepoint cases, Patient 2 (G) is a grade 4 astrocytoma that demonstrated an increase in nrCBV and a decrease in ADC after day 117 of AG-120 treatment and presented with a PFS of around 173 days. Patient 3 (H) is a grade 3 astrocytoma who exhibited a small decrease in nrCBV and small increase in ADC after day 112 of AG-120 treatment. Patient 3 had a longer PFS of 1121 days. IDH, isocitrate dehydrogenase; nrCBV, normalized relative cerebral blood volume; PFS, progression-free survival.

To assess how imaging measurements changed over time between patients exhibiting long versus short PFS, we pooled cross-sectional nrCBV, ADC, and FLAIR volume measurements in all patients and time points and used polynomial regression to visualize possible trends (Figure 2A–C). While not significantly different, results show an intuitive early increase in FLAIR volume in the cohort of patients with a short PFS and a relatively flat trajectory in FLAIR volume within the cohort of patients exhibiting longer PFS. Results also suggest a transient increase in nrCBV may be observed shortly after starting treatment, but a sustained increase in nrCBV over time is observed in patients with a shorter PFS. Additionally, patients with a long PFS had relatively stable ADC, whereas a continuous decrease in ADC was seen in patients with shorter PFS. These trends are also illustrated in three representative cases of IDH1-mutant glioma patients with differential responses to IDH inhibition (Figure 2D–H). The first patient exhibited a large, early increase in nrCBV at the early 3–6-week timepoint after treatment and presented with a PFS of around 197 days (Figure 2F). Patient 2 demonstrated an increase in nrCBV and a decrease in ADC at the later 2–4-month timepoint and presented with a PFS of around 173 days (Figure 2G). Meanwhile, patient 3 exhibited a small decrease in nrCBV and a small increase in ADC at the later 2–4-month timepoint and presented with a PFS of around 1121 days (Figure 2H).

**Figure 3.**
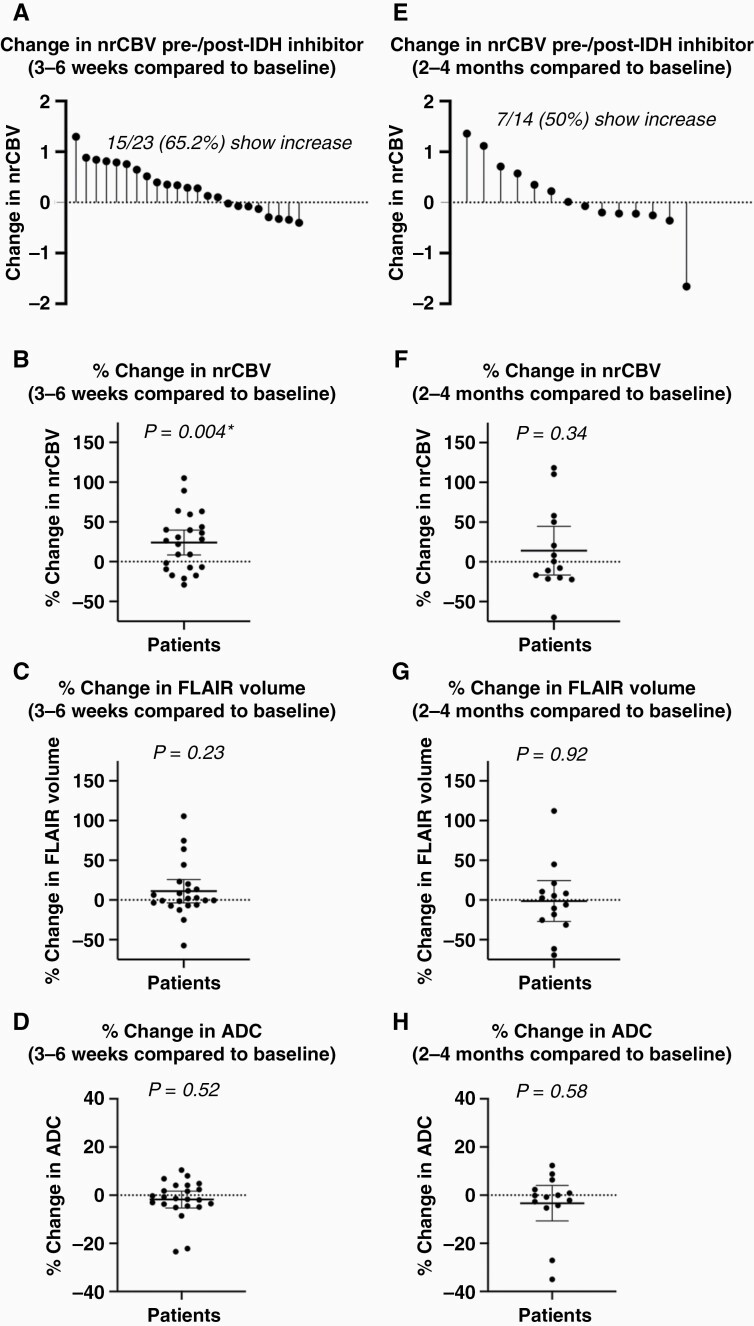
Quantitative comparison of changes in nrCBV, FLAIR volume, and ADC after IDH inhibitor treatment. After 3–6 weeks of IDH inhibitor treatment, there was (A, B) a significant increase in nrCBV (*P *= .004) (C) and no change in FLAIR volume (*P* = .23) (D) or ADC (*P *= .52). After 2–4 months of IDH inhibitor treatment, (E, F) nrCBV, (G) FLAIR volume, and (H) ADC appeared to stabilize. Asterisks (*) indicate *P* < .05. IDH, isocitrate dehydrogenase; nrCBV, normalized relative cerebral blood volume; ADC, apparent diffusion coefficient.

**Figure 4.**
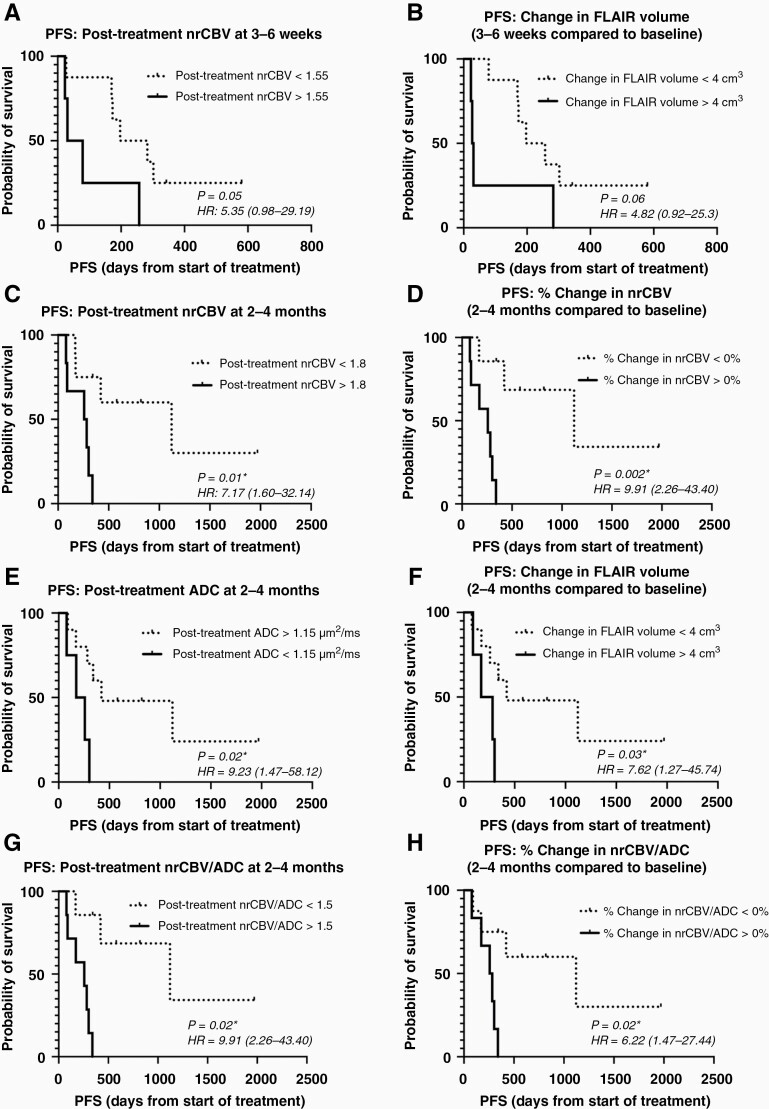
Survival curves displaying relationships between nrCBV, ADC, FLAIR volume, and PFS. (A) Relationship between PFS and nrCBV values at 3–6 weeks of treatment (*P* = .05). (B) Relationship between PFS and change in FLAIR volume at 3–6 weeks of treatment compared to baseline (*P* = 0.06). (C) Relationship between PFS and nrCBV values at 2–4 months of treatment (*P* = .01). (D) Relationship between PFS and % change in nrCBV values at 2–4 months of treatment compared to baseline (*P* = .002). (E) Relationship between PFS and posttreatment ADC values at 2–4 months of treatment (*P* = .02). (F) Relationship between PFS and change in FLAIR volume at 2–4 months compared to baseline (*P* = .03). (G) Relationship between PFS and median nrCBV/ADC ratios at 2–4 months of treatment (*P* = .002). (H) Relationship between PFS and % change in median nrCBV/ADC ratios at 2–4 months of treatment compared to baseline (*P* = .02). Asterisks (*) indicate *P *< .05. PFS, progression-free survival; nrCBV, normalized relative cerebral blood volume; ADC, apparent diffusion coefficient.

Consistent with these visual trends, there was an overall significant increase in nrCBV (Figure 3A and B; one-sample *t*-test; *P* = .004; mean %change = 24.15% [95% CI: 8.52 to 39.79]) and in median nrCBV/ADC ratio (Wilcoxon Signed-Rank test; *P* = .003; mean %change = 28.51% [8.84 to 48.18]) 3–6 weeks after start of treatment, while there was no change in FLAIR volume (Figure 3C; Wilcoxon Signed-Rank test; *P* = .23; mean %change = 11.05% [−3.70 to 25.80)] or ADC (Figure 3D; Wilcoxon Signed-Rank test; *P* = .52; mean %change = −1.77% [−5.28 to 1.73]). A total of 15 of 23 patients (65.2%) exhibited an increase in nrCBV at this early time point; however, high posttreatment nrCBV > 1.55 only *trended* toward shorter PFS by 3–6 weeks posttreatment (Figure 4A; Log-Rank test; *P* = .05; HR = 5.35 [0.98 to 29.19]; median PFS = 240 days vs 55 days). High posttreatment median nrCBV/ADC ratio > 1.20 yielded the same results as posttreatment nrCBV > 1.55 with shorter PFS (Supplementary Figure S1A; Log-Rank test; *P* = .05; HR = 5.35 [0.98 to 29.19]; median PFS = 240 days vs 55 days). Additionally, increase in FLAIR volume > 4 cm^3^ at 3–6 weeks also trended toward shorter PFS (Figure 4B; Log-Rank test; *P* = .06; HR = 4.82 [0.92 to 25.30]; median PFS = 227 days vs 29 days), while the percentage change in FLAIR volume did not show a significant association with PFS. Posttreatment ADC, percentage change in ADC, and percentage change in nrCBV were not significantly associated with PFS.

At 2–4 months of treatment, there was no significant change in nrCBV (one-sample *t*-test; *P* = .34; mean %change = 14.08% [−16.49 to 44.65]), ADC (Wilcoxon Signed-Rank test; *P* = .58; mean %change = −3.34 [−10.73 to 4.06]), median nrCBV/ADC ratio (Wilcoxon Signed-Rank test; *P* = .63; mean %change = 25.42% [−17.05 to 67.88]), or FLAIR volume [one-sample *t*-test; *P* = .92; mean %change = 1.27% [−27.06 to 24.52]) relative to baseline measurements (Figure 3E–H). At this timepoint, posttreatment nrCBV > 1.80 (Figure 4C; Log-Rank test; *P* = .01; HR = 7.17 [1.60 to 32.14]; median PFS = 1121 days vs 270 days), increase in nrCBV > 0% compared to baseline (Figure 4D; Log-Rank test; *P* = .002; HR = 9.91 (2.26 to 43.40); median PFS = 1121 days vs 257 days), posttreatment ADC < 1.15 μm^2^/ms (Figure 4E; Log-Rank test; *P* = .02; HR = 9.23 [1.47 to 58.12]; median PFS = 421 days vs 215 days), change in ADC < −2% compared to baseline (Supplementary Figure S1B; Log-Rank test; *P* = .01; HR = 7.32 [1.63 to 32.94]; median PFS = 1121 days vs 228 days), increased FLAIR volume > 4cm^3^ (Figure 4F; Log-Rank test; *P* = .03; HR = 7.62 [1.27 to 45.74]; median PFS = 421 vs 226.5 days), posttreatment median nrCBV/ADC ratio > 1.50 (Figure 4G; Log-Rank test; *P* = .002; HR = 9.91 [2.26 to 43.40]; median PFS = 1121 days vs 257 days; same results as increase in nrCBV > 0%), and increase in median nrCBV/ADC ratio > 0% compared to baseline (Figure 4H; Log-Rank test; *P* = .02; HR = 6.22 [1.41 to 27.44]; median PFS = 1121 vs 270 days) were significantly associated with shorter PFS.

Univariate Cox regression analysis for PFS was significant when considering continuous measures of percentage change in nrCBV (*P* = .02), percentage change in ADC (*P* = .04), posttreatment median nrCBV/ADC ratio (*P* = .04), and percentage change in median nrCBV/ADC ratio (*P* = .01) at 2–4 months from initiation of IDH inhibitor treatment while posttreatment nrCBV (*P* = .09) and posttreatment ADC (*P* = 0.06) *trended* toward significance (Table 2). After accounting for patient age and tumor grade, percentage change in nrCBV (Table 2; Multivariate Cox regression; *P* = .03), posttreatment median nrCBV/ADC ratio (*P* = .03), and percentage change in median nrCBV/ADC ratio (*P* = .03) remained significant.

**Table 2.** Univariate and Multivariate Cox Regression Results for PFS Controlling for Patient Age and Tumor Grade Using Continuous Measures of MRI Metrics

MRI Metrics	PFS (Univariate)			PFS (Multivariate)		
2–4 Months	HR	*Z*-value	*P*-value	HR	*Z*-value	*P*-value
Posttreatment nrCBV	2.20 (0.89–5.45)	1.71	.09	N/A		
%Change in nrCBV	1.02 (1.00–1.03)	2.34	.02*	1.02 (1.00–1.04)	2.18	.03*
Posttreatment ADC	0.99 (0.99–1.00)	−1.89	.06	N/A		
%Change in ADC	0.95 (0.90–0.998)	−2.04	.04*	0.96 (0.91–1.01)	–1.64	.10
Posttreatment median nrCBV/ADC ratio	3.26 (1.05–21.95)	2.04	.04*	3.77 (1.17–12.14)	2.22	.03*
%Change in median nrCBV/ADC ratio	1.02 (1.00–1.03)	2.57	.01*	1.02 (1.00–1.03)	2.17	.03*
Change in FLAIR volume	0.99 (0.97–1.01)	−1.25	.21	N/A		

*Note*: Asterisks (*) indicate *P *< .05.

Abbreviation: PFS, progression-free survival; nrCBV, normalized relative cerebral blood volume; ADC, apparent diffusion coefficient; FLAIR, fluid-attenuated inversion recovery; HR, hazard ratio.

## Discussion

Results from the current study provide the first evidence that early, transient changes in tumor vascularity may occur following IDH inhibition in human IDH1-mutant gliomas. Results suggest a large proportion (~65%) of patients exhibit early increases in nrCBV within 3–6 weeks following treatment initiation, but by 2–4 months from treatment initiation far fewer and less substantial changes in nrCBV compared to baseline occur. While early, elevated posttreatment nrCBV *trended* toward shorter PFS by 3–6 weeks after the start of treatment, changes in nrCBV and median nrCBV/ADC ratio at 2–4 months after treatment were strong predictors of long-term PFS. Additionally, increases in FLAIR volume greater than 4 cm^3^ at 3–6 weeks and 2–4 months from the start of treatment were associated with shorter PFS. Finally, decreases in ADC and low ADC at 2–4 months from the start of treatment were suggestive of lower PFS.

The biological mechanisms underpinning these transient and impactful changes in vascularity and tumor size are not well understood. It is possible that the initial increase in tumor volume and nrCBV at 3–6 weeks may reflect competing effects of continued tumor growth and therapeutic response early in the treatment course. To our knowledge, this study is the first to assess MRI changes following IDH inhibition as early as 3–6 weeks following initial treatment, so previous clinical studies describing tumor shrinkage in glioma patients^19,20^ may not have taken into consideration these early, transient changes. Nevertheless, patients on average exhibited at least a small increase in tumor volume at 3–6 weeks, which was associated with more favorable response compared with patients exhibiting more substantial increases over the same period, supporting the idea of a potential mixed response early after treatment.

Additionally, the early increase in nrCBV may reflect transiently increased vascularity or vascular volume. While it is possible that this early rise in nrCBV in the present study may also be from continued tumor growth, we speculate that this observation may reflect downstream alterations caused by decreased D-2-HG levels, which could have led to elevated HIF-1α levels and promote increased hypoxic and subsequent proangiogenic signaling.^12,13,45^ Importantly, this early rise in nrCBV and tumor volume was no longer observed after 2–4 months of treatment, and there was a PFS benefit in patients who exhibited less elevated nrCBV values or < 0% change in nrCBV at this later timepoint. Although speculative, the apparent stabilization of nrCBV after 2–4 months of treatment in patients who had a longer PFS may reflect a useful timepoint to assess IDH inhibition using perfusion MRI.

It is also important to note that no significant changes in ADC were observed at either posttreatment timepoint compared to baseline but decreases in ADC at 2–4 months were associated with poorer PFS. As ADC is thought to be inversely proportional to tumor cell density,^46^ this finding is consistent with the work presented by Molloy et al.^21^ where they observed no change in the cell density in genetically engineered IDH1-mutated cell lines after IDH inhibition, suggestive of no alteration in cell death or proliferation rate. In patients with AML, IDH inhibitors act as cellular differentiation agents, not as cytotoxic agents.^47^ Mutant IDH inhibition in glioma cells has also been demonstrated to promote differentiation.^22^ The lack of observed changes in ADC in the current study appears to support the hypothesis that IDH inhibition may not have a strong cytotoxic effect in gliomas and that the therapeutic effect of this treatment may be the result of glioma cell differentiation. Meanwhile, the decrease in ADC being associated with reduced PFS at the later 2–4-month timepoint is likely a reflection of continued tumor progression and treatment resistance. Furthermore, the ratio of median nrCBV/ADC was the only metric at 2–4 months of treatment where both posttreatment values and percentage change remained significant after accounting for tumor grade and age. Previous studies have concurrently examined perfusion and diffusion characteristics of gliomas,^48^ including median nrCBV/ADC ratios.^42^ The present findings suggest that combined perfusion and diffusion metrics may be valuable in evaluating IDH inhibition given the individual associations of high nrCBV and low ADC with reduced PFS. While largely speculative, future studies aimed at more thoroughly documenting longitudinal changes in anatomic, physiologic, and metabolic MRI are warranted to better understand the temporal changes that occur after IDH inhibition in human IDH1-mutant gliomas.

There are several limitations in this study that should be addressed. First, the sample size in the current study was relatively small and derived from a single center consisting of a relatively heterogenous sample of patients. Because of the indolent nature of IDH-mutant gliomas, clinical trials of IDH inhibitors in patients with IDH-mutant gliomas have also included patients with recurrent gliomas who received prior therapies.^19,20^ Furthermore, the small study cohort involved patients with various tumor grades and 1p/19q co-deletion status. While multivariate Cox regression analysis yielded significant results while controlling for tumor grade, 1p/19q co-deletion status was unable to be included as a covariate because 1p/19q co-deletion status was unavailable for some patients, and dichotomization of our patient population based on 1p/19q status would have further reduced our limited sample size. It is possible that 1p/19q co-deletion status may be a confound in the present findings that warrant further investigation. Increasing the sample size would also be valuable to perform multiple comparisons corrections on the present study’s findings. Moreover, given the retrospective nature of this study and off-label use of IDH inhibitors in some patients, it was not possible to control for the time interval between scans and the usage of other concurrent treatments. Importantly, one patient received concurrent bevacizumab during IDH inhibitor treatment, but, likely, this did not confound our results because they exhibited a rise in nrCBV during the course of this study, even though anti-angiogenic therapy response should cause a notable decrease in tumor volume and nrCBV.^30^ Additionally, overall survival was unable to be tested given the relatively long overall survival of patients with IDH1-mutant gliomas and the only recent usage of IDH inhibitors in patients, which would result in a significant number of censored patients in the present study cohort. Finally, it is important to note that the associations of perfusion, diffusion, and volumetric MRI metrics with PFS in this limited patient cohort are not reflective of any potential benefit of IDH inhibitor therapy (see commentary on survival by tumor response by Anderson and Gelber^49^) in IDH1-mutant gliomas, but simply reflect patient stratifications based on radiographic assessment within patients treated with IDH inhibitors. As a result, future studies with a larger study cohort that can further assess the associations of age, tumor grade, 1p/19q co-deletion status, glioma recurrence status, contrast enhancement, prior treatments, and overall survival would be valuable to better understand the findings of this present study and, more broadly, add to the present study’s findings on interpreting the radiographic response to IDH inhibitors in patients with IDH1-mutant gliomas.

## Conclusion

The current pilot study demonstrated a transient increase in perfusion that appears to stabilize after 2–4 months, at which changes in perfusion and ADC relative to baseline were strongly associated with resulting PFS. Results suggest that FLAIR volume, nrCBV, and ADC measurements may be useful early imaging biomarkers for assessing IDH inhibitor treatment response in human IDH1-mutant gliomas.

## Supplementary Material

vdac124_suppl_Supplementary_Material

## Prior Presentation

A portion of this study was presented at the 2022 American Society of Neuroradiology Annual Meeting.
