# Supplementary material for: Early volumetric, perfusion, and diffusion MRI changes after mutant isocitrate dehydrogenase (IDH) inhibitor treatment in IDH1-mutant gliomas
Source: Neurooncol Adv. 2022 Aug 4;4(1):vdac124. doi: 10.1093/noajnl/vdac124 (PMC9400453; doi:10.1093/noajnl/vdac124)
Supplement: vdac124_suppl_Supplementary_Material [file vdac124_suppl_supplementary_material.docx]

Early Volumetric, Perfusion, and Diffusion MRI Changes after Mutant Isocitrate Dehydrogenase (IDH) Inhibitor Treatment in IDH1-Mutant Gliomas

Nicholas S. Cho^1-4^, Akifumi Hagiwara^2,3,5^, Blaine S.C. Eldred^6,7^, Catalina Raymond^2,3^, Chencai Wang^2,3^, Francesco Sanvito^2,3,8^, Albert Lai^6,7^, Phioanh Nghiemphu^6,7^, Noriko Salamon^3^,

Lori Steelman^9^, Islam Hassan^9^, Timothy F. Cloughesy^6,7^, Benjamin M. Ellingson^2-4,10,11*^

^1^ Medical Scientist Training Program, David Geffen School of Medicine, University of California, Los Angeles, Los Angeles, CA, USA.

^2^ UCLA Brain Tumor Imaging Laboratory (BTIL), Center for Computer Vision and Imaging Biomarkers, University of California, Los Angeles, Los Angeles, CA, USA

^3^ Department of Radiological Sciences, David Geffen School of Medicine, University of California, Los Angeles, Los Angeles, CA, USA

^4^ Department of Bioengineering, Henry Samueli School of Engineering and Applied Science, University of California Los Angeles, Los Angeles, CA, USA

^5^ Department of Radiology, Juntendo University School of Medicine, Tokyo, Japan

^6^ UCLA Neuro-Oncology Program, David Geffen School of Medicine, University of California, Los Angeles, Los Angeles, CA, USA

^7^ Department of Neurology, David Geffen School of Medicine, University of California, Los Angeles, Los Angeles, CA, USA

^8^ Unit of Radiology, Department of Clinical, Surgical, Diagnostic, and Pediatric Sciences, University of Pavia, Pavia, Italy

^9^ Servier Pharmaceuticals, LLC, Boston, Massachusetts, USA

^10^ Department of Neurosurgery, David Geffen School of Medicine, University of California, Los Angeles, Los Angeles, CA, USA

^11^ Department of Psychiatry and Biobehavioral Sciences, David Geffen School of Medicine, University of California, Los Angeles, Los Angeles, CA, USA

**Running Title:** Perfusion and Diffusion MRI after IDH inhibition

***Corresponding author**

Benjamin M. Ellingson, Ph.D.

Director, UCLA Brain Tumor Imaging Laboratory (BTIL)

Professor of Radiology, Psychiatry, and Neurosurgery

Departments of Radiological Sciences, Psychiatry, and Neurosurgery

David Geffen School of Medicine

University of California, Los Angeles

924 Westwood Blvd., Suite 615,

Los Angeles, CA 90024 (bellingson@mednet.ucla.edu).

Phone: 310-481-7572

Fax: 310-794-2796

**Supplementary Table 1. Patient IDH Inhibitor Treatment Information**

| **ID** | **Sex** | **Age** | **IDH Inhibitor** | **Dosing (Daily)** |
| --- | --- | --- | --- | --- |
| 1 | M | 62 | AG-120 | 600 mg |
| 2 | M | 51 | AG-120 | 250 mg for first 2 weeks, then 500 mg |
| 3 | F | 48 | AG-120 | 500 mg |
| 4 | F | 65 | AG-881 | 10 mg |
| 5 | F | 51 | AG-120 | 500 mg |
| 6 | M | 38 | AG-120 | 500 mg |
| 7 | M | 47 | AG-881 | 25 mg |
| 8 | M | 33 | AG-120 | 500 mg |
| 9 | F | 34 | AG-881 | 300 mg |
| 10 | M | 48 | AG-991 | 50 mg |
| 11 | F | 63 | AG-120 | 500 mg |
| 12 | M | 59 | AG-120 | 500 mg |
| 13 | M | 43 | AG-881 | 10 mg |
| 14 | F | 40 | AG-881 | 100 mg for first 3 months, then 200 mg |
| 15 | F | 50 | AG-881 | 100 mg |
| 16 | M | 27 | AG-120 | 500 mg |
| 17 | M | 25 | AG-120 | 500 mg |
| 18 | M | 33 | AG-120 | 500 mg |
| 19 | F | 19 | AG-881 | 10 mg |
| 20 | F | 37 | AG-120 | 500 mg |
| 21 | M | 25 | AG-120 | 500 mg |
| 22 | M | 43 | AG-881 | 200 mg |
| 23 | M | 45 | AG-120 | 500 mg |
| 24 | F | 39 | AG-120 | 500 mg |
| 25 | M | 40 | AG-881 | 50 mg |
| 26 | M | 34 | AG-120 | 500 mg |
| 27 | F | 52 | AG-881 | 50 mg |
| 28 | M | 29 | AG-120 | 500 mg |
| 29 | M | 32 | AG-120 | 500 mg |

**Supplementary Figure 1.**


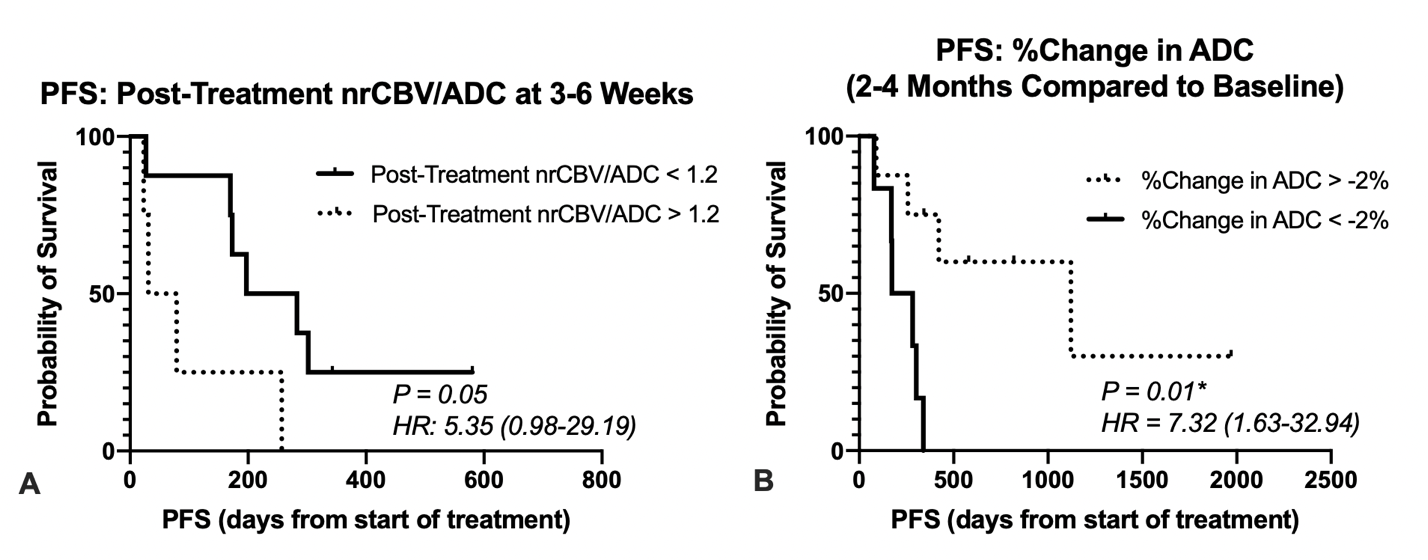
**Figure S1.** Survival curves displaying relationships between nrCBV/ADC and ADC with PFS. **(A)** Relationship between PFS and median nrCBV/ADC ratios at 3–6 weeks (*P* = 0.05). **(B)** Relationship between PFS and percentage change in ADC at 2–4 months (*P* = 0.01). Asterisks (*) indicate *P* < 0.05. PFS = progression-free survival; nrCBV = normalized relative cerebral blood volume; ADC = apparent diffusion coefficient
